# A “footprint” of plant carbon fixation cycle functions during the development of a heterotrophic fungus

**DOI:** 10.1038/srep12952

**Published:** 2015-08-11

**Authors:** Xueliang Lyu, Cuicui Shen, Jiatao Xie, Yanping Fu, Daohong Jiang, Zijin Hu, Lihua Tang, Liguang Tang, Feng Ding, Kunfei Li, Song Wu, Yanping Hu, Lilian Luo, Yuanhao Li, Qihua Wang, Guoqing Li, Jiasen Cheng

**Affiliations:** 1 State Key Laboratory of Agricultural Microbiology, Huazhong Agricultural University, Wuhan 430070, Hubei Province, China; 2 The Provincial Key Lab of Plant Pathology of Hubei Province, College of Plant Science and Technology, Huazhong Agricultural University, Wuhan 430070, Hubei Province, China

## Abstract

Carbon fixation pathway of plants (CFPP) in photosynthesis converts solar energy to biomass, bio-products and biofuel. Intriguingly, a large number of heterotrophic fungi also possess enzymes functionally associated with CFPP, raising the questions about their roles in fungal development and in evolution. Here, we report on the presence of 17 CFPP associated enzymes (ten in Calvin-Benson-Basham reductive pentose phosphate pathway and seven in C4-dicarboxylic acid cycle) in the genome of *Sclerotinia sclerotiorum*, a heterotrophic phytopathogenic fungus, and only two unique enzymes: ribulose-1, 5-bisphosphate carboxylase-oxygenase (Rubisco) and phosphoribulokinase (PRK) were absent. This data suggested an incomplete CFPP-like pathway (CLP) in fungi. Functional profile analysis demonstrated that the activity of the incomplete CLP was dramatically regulated during different developmental stages of *S. sclerotiorum*. Subsequent experiments confirmed that many of them were essential to the virulence and/or sclerotial formation. Most of the CLP associated genes are conserved in fungi. Phylogenetic analysis showed that many of them have undergone gene duplication, gene acquisition or loss and functional diversification in evolutionary history. These findings showed an evolutionary links in the carbon fixation processes of autotrophs and heterotrophs and implicated the functions of related genes were in course of continuous change in different organisms in evolution.

Carbon fixation is one of the key to categorize organisms into autotrophs and heterotrophs. To deepen the understanding of the evolutionary links in the carbon fixation processes of autotrophs and heterotrophs, it is essential to compare their carbon metabolism networks and associated genes. Meanwhile, carbon fixation in photosynthesis or human industrial engineering is essential to satisfy the increased demand for bio-products, biofuel or sustainable energy sources. However, photosynthetic carbon assimilation is one of the bottlenecks in photosynthesis which is the only major natural solar energy storage mechanism on earth^1^. The efficiency of carbon fixation is one of many challenges that constrain the product yields in modern fermentation engineering. These reasons promote the research on the evolution and biological function of carbon fixation pathway. Carbon fixation in photosynthesis is the reduction of inorganic carbon (carbon dioxide) to organic compounds by autotrophic plants, which is fundamental for the existence and evolution of biosphere^2^,^3^. There are multiple carbon fixation pathways, and the C3 and C4 carbon fixation pathways are the most common ones in land plants, especially crops. C3 plants and cyanobacteria fix CO_2_ into biomass by the Calvin-Benson-Basham (CBB) cycle which is the predominant mechanism of carbon fixation. With an additional C4-dicarboxylic acid cycle, C4 plants have an advantage over C3 plants under the condition of CO_2_ limitation and hence have an enhanced ability to fix carbon^4^,^5^.

Previously, some fungi were found to have an ability to fix carbon dioxide^6^,^7^,^8^, among which some could assimilate CO_2_ into oxaloacetate, a precursor of oxalic acid^6^. However, whether carbon fixation pathways exist in heterotrophic organisms is not clearly known. Analysis of fungal genomes showed that fungi do not have homologs of ribulose-1, 5-bisphosphate carboxylase-oxygenase (RuBisCO) and phosphoribulokinase (PRK) in the CBB cycle. However, recently functional expression of the heterogenous PRK and Rubisco in *Saccharomyces cerevisiae* could enabled the use of CO_2_ as electron acceptor for NADH oxidation, and hence increased the ethanol yield in industrial biotechnology^9^, indicating fungi have the potential to complete carbon fixation pathway, which could be activated by the supplement of a few key enzymes.

*Sclerotinia sclerotiorum*, a member of Ascomycota, is a fungal plant pathogen with a worldwide distribution and remarkably broad range of hosts. At least 408 described species of plants ranging from 278 genera in 75 families are susceptible to this pathogen^10^,^11^. Diseases caused by *S. sclerotiorum* are known as white mold, Sclerotinia stem rot, wilt or stalk rot and Sclerotinia head rot, and often lead to significant losses every year due to lack of adequately resistant cultivars^11^. There are six key steps in the life cycle of *S. sclerotiorum*, namely vegetative growth, infection, sclerotial formation, myceliogenic germination of sclerotia, carpogenic germination of sclerotia and sexual reproduction. *S. sclerotiorum* is a typical necrotrophic pathogen, it secretes oxalic acid to disarm host resistance and kill host cells and tissues^11^,^12^,^13^. Like other necrotrophic fungal plant pathogens, *S. sclerotiorum* also produces many cell-wall-degrading enzymes to facilitate the penetration and colonization of its hosts^11^. Sclerotia, dormant bodies of *S. sclerotiorum*, are very important for its survival in nature, sclerotia may germinate to produce infectious hyphae directly, or germinate carpogenically to produce ascospores in apothecia^11^. The release of the genome sequence of *S. sclerotiorum* has immensely facilitated our understanding of this fungal pathogen^14^.

In this study, Illumina next-generation sequencing technology was used to analyze the transcriptome of *S. sclerotiorum* in response to hyphal growth on potato dextrose agar (PDA) medium, the early stage infection on *Arabidopsis thaliana*, sclerotial development, sclerotial myceliogenic germination, sclerotial carpogenic germination and apothecium formation (stipe). Intriguingly, our analysis demonstrated that many enzymes functionally associated to CFPP were dramatically regulated during the development of *S. sclerotiorum*. Subsequent functional experiments demonstrated their crucial roles during the infection and (or) sclerotial development. Taken together, we provide an example that fungi possess genes functionally homologous to CFPP-associated genes in both the CBB cycle and the C4-dicarboxylic acid cycle, which are crucial for fungal virulence and development.

## Results

### The functional profile analysis and gene functional enrichment analysis

The digital gene expression (DGE) system was initially used to gain an insight into the wide range of transcriptional responses associated with vegetative growth, infection, sclerotial development, myceliogenic germination of sclerotia, carpogenic germination of sclerotia and apothecium formation of *S. sclerotiorum*. In total, the six representative cDNA libraries produced more than 20.6 million tags. In each library, a sequencing depth of approximately 3.3 million clean tags was obtained after the removal of potentially erroneous tags (Table 1). All of the clean tags were mapped to the predicted reference transcripts in Broad Institute database (http://www.broadinstitute.org/). The number of the detected tag-mapped genes ranged from 6901 to 8142 in the six cDNA libraries (Table 1). Finally, 66.7% (9,672/14,503) of the reference genes could be mapped (Table S1). To globally understand the dynamic change of various gene functional modules on different levels during *S. sclerotiorum* development, a method namely “functional profile analysis” was developed according to the measure of expression levels of gene ontology (GO) categories^15^. “Functional profile analysis” combined the gene expression level with the gene functional annotation (Table S2) to illustrate the activity of corresponding functional modules. Through the comparison of “functional profiles”, we could detect the dynamic change of the relative activity of various functional modules on different levels during different developmental stages of *S. sclerotiorum*. In addition, a gene functional enrichment analysis was performed to detect the significantly over-represented functional modules during corresponding developmental stages. As expected, our analysis indicated multiple functional modules play synergistic roles during different developmental stages of *S. sclerotiorum.* For example, functional profile analysis demonstrated functional modules associated with cell redox homeostasis was dramatically increased during all of the developmental stages compared to that during the vegetative growth stage (Figure S1 and Table S3), and some of the associated functional modules (such as “response to oxidative stress”, “peroxidase activity”, “antioxidant activity”, “oxidase”-related and “dehydrogenase”-related molecular modules) were also over-represented during corresponding developmental stages by the gene functional enrichment analysis (Figure S2 and Table S5). On the contrary, our functional profile analysis suggested that the translation associated processes were dramatically decreased during all of the developmental stages compared to that during the vegetative growth stage (Figure S1 and Table S3), this result was also consistent with the gene functional enrichment analysis which indicated that most of the enriched translation associated functional modules (such as “structural constituent of ribosome”) were under-represented during corresponding stages (Figure S2 and Table S5). In addition, the functional profile analysis and the gene functional enrichment analysis demonstrated many KEGG metabolic pathways were involved in multiple developmental processes of *S. sclerotiorum* (Table S4 and S6). More details of the functional profile analysis and gene functional enrichment analysis see the table S3, S4, S5 and S6. Specially, our functional profile analysis showed an incomplete CFPP-like pathway (CLP) was also dramatically activated during the infection, sclerotial myceliogenic germination and sclerotial carpogenic germination of *S. sclerotiorum* (Table S4), and this CLP was selected for further study.

### The significant regulation of CLP associated genes during different developmental stages of *S. sclerotiorum*

**Figure 1:**
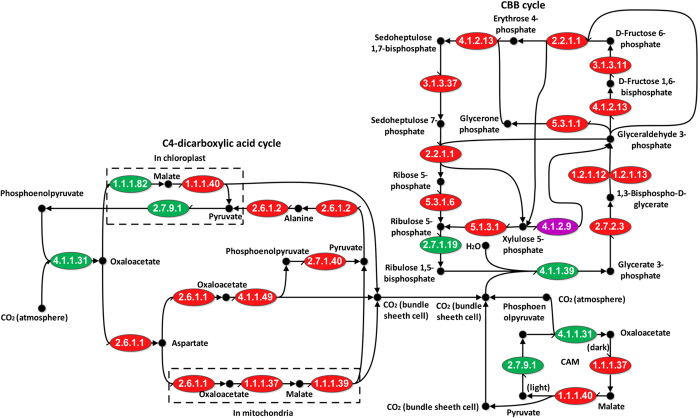
Fungal genes mapped to the carbon fixation pathway according to the path: map00710 in KEGG database. Enzyme commission (EC) codes in red ellipses indicate the corresponding enzymes are present in both *S. sclerotiorum* and *A. thaliana*. EC codes in green ellipses indicate the corresponding enzymes are present only in *A. thaliana*. EC code in purple ellipse indicate the corresponding enzyme is present only in *S. sclerotiorum*. Solid black dots indicate the compounds or intermediate products in the carbon fixation pathway.

A common CFPP map was created according to the reference pathway in KEGG database (path: map00710)^16^ with 22 enzymes being involved in the carbon fixation of C3 or C4 plants, including 12 enzymes in the CBB cycle and ten enzymes in the C4-dicarboxylic acid cycle. This pathway also includes four enzymes in crassulacean acid metabolism (CAM) pathway in Crassulaceae plant family and one special enzyme (phosphoketolase, EC: 4.1.2.9) that play roles in the carbon fixation of some prokaryotes (Fig. 1). Ten CBB cycle associated enzymes were found in *S. sclerotiorum* genome. These enzymes are ribose-5-phosphate isomerase (EC: 5.3.1.6, SS1G_08408), triose-phosphate isomerase (EC: 5.3.1.1, SS1G_11433), ribulose-phosphate 3-epimerase (EC: 5.1.3.1, SS1G_01844), transketolase (EC: 2.2.1.1, SS1G_10246), phosphoglycerate kinase (EC: 2.7.2.3, SS1G_01105), fructose-bisphosphate aldolase (EC: 4.1.2.13, SS1G_06561 and SS1G_06400), fructose-bisphosphatase or sedoheptulose-bisphosphatase (EC: 3.1.3.11 or EC: 3.1.3.37, SS1G_11369) and glyceraldehyde-3-phosphate dehydrogenase (phosphorylating or NADP^+^, phosphorylating) (EC: 1.2.1.12 or EC: 1.2.1.13, SS1G_07798). Only two genes, Rubisco (EC: 4.1.1.39) and PRK (EC: 2.7.1.19) in the CBB cycle, have never been found in *S. sclerotiorum*. In addition, seven C4-dicarboxylic acid cycle associated enzymes were found in *S. sclerotiorum*. These enzymes are phosphoenolpyruvate carboxykinase (ATP) (EC: 4.1.1.49, SS1G_02281), malate dehydrogenase (EC: 1.1.1.37, SS1G_08975 and SS1G_13825), alanine transaminase (EC: 2.6.1.2, SS1G_02202), aspartate transaminase (EC: 2.6.1.1, SS1G_03827 and SS1G_14097), pyruvate kinase (EC: 2.7.1.40, SS1G_04568), malate dehydrogenase (oxaloacetate-decarboxylating, NADP^+^) and malate dehydrogenase (decarboxylating) (EC: 1.1.1.40 and EC: 1.1.1.39, SS1G_08827 and SS1G_12079) in *S. sclerotiorum*. Interestingly, two *S. sclerotiorum* genes (*SS1G_06022* and *SS1G_06790*) encoding the phosphoketolase (EC: 4.1.2.9) which play roles in carbon fixation of some prokaryotes were also present in the path: map00710 reference pathway. The fungal genes and their corresponding homologs in *A. thaliana* are listed in Table 2, and the predicted conserved domains of these fungal enzymes related to plant carbon fixation are listed in Table S2. The fungal enzymes and their positions were mapped to the common CFPP pathway (Fig. 1). In conclusion, there is an incomplete carbon fixation pathway which is similar to CFPP in *S. sclerotiorum*, and it is designated as CLP.

In addition, our DGE data suggested that 16 of the 20 fungal CLP associated genes were significantly regulated during different developmental stages. To validate our DGE data, the 20 fungal CLP associated genes were selected for quantitative reverse transcription PCR (qRT-PCR) analysis. The data was presented as fold changes in gene expression normalized to the β-tubulin gene^17^,^18^,^19^. Pearson’s correlation coefficient (R value) was used to measure the consistency of the qRT-PCR data and DGE data. The R values showed most of the qRT-PCR results were strongly correlated with the DGE data (Figure S3), which indicated that the expression patterns of most of the 20 genes in our DGE data and qRT-PCR data were accordant. These results indicated the reliability of our DGE data for transcription analysis. According to the DGE data, there were nine and three genes were significantly up-regulated and down-regulated respectively during the infection, compared to the vegetative growth stage. Six genes were significantly induced during the sclerotial development stage (Table 3). Taken together, our results suggested the fungal CLP associated genes may play important roles during different developmental stages of *S. sclerotiorum*.

### RNAi mediated down-regulation of fungal CLP associated genes impaired the virulence and (or) sclerotial development of *S. sclerotiorum*

**Figure 2:**
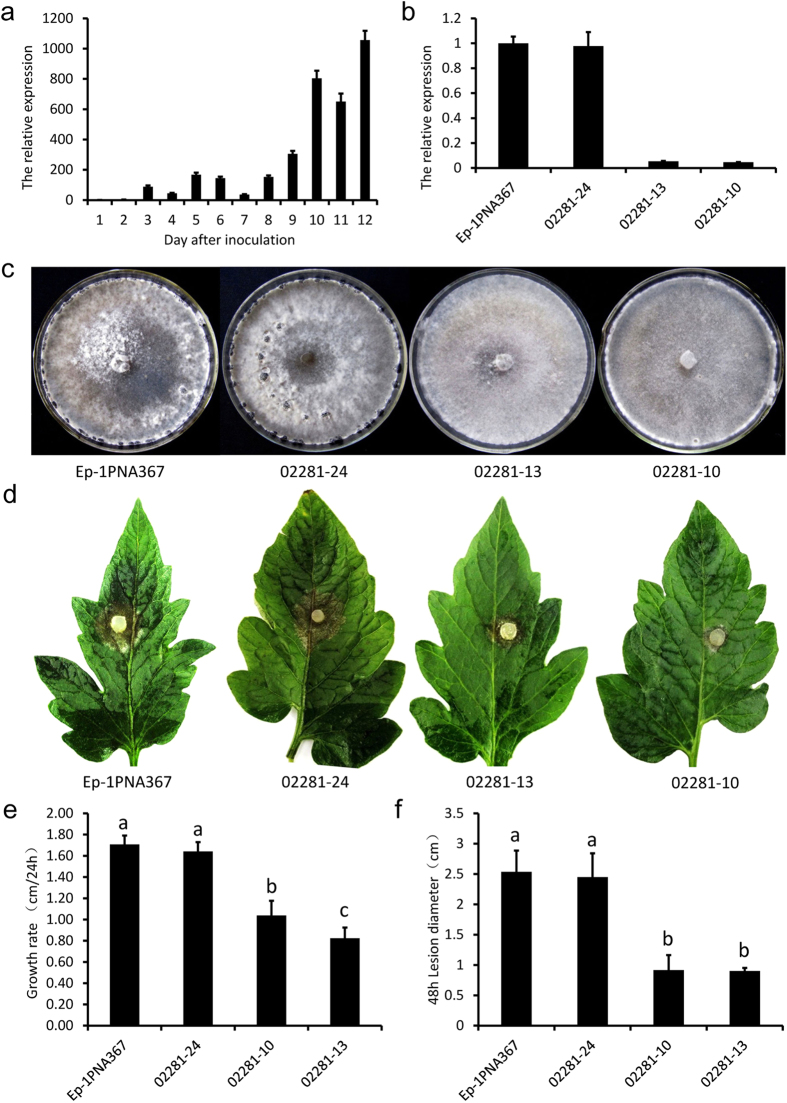
Biological characterization of the RNAi-silenced transformants of a fungal CLP associated gene (*SS1G_02281*) encoding for phosphoenolpyruvate carboxykinase (ATP). (**a**). QRT-PCR analysis of *SS1G_02281* gene transcripts from the first day to the twelfth day. Wild-type strain Ep-1PNA367 was cultured on PDA and mycelial mass (including sclerotia) was collected every day for 1–12 days. A value of 1 was assigned to the abundance of *SS1G_02281* transcripts from the first day’s mycelia mass. (**b**). Relative expression level of *SS1G_02281* gene in RNAi-silenced transformants and wild-type strain determined with qRT-PCR analysis. RNA samples were extracted from mycelia mass collected from colony growing on PDA for 12 days. A value of 1 was assigned to the abundance of cDNA from the wild-type strain. The gene expression levels of RNAi-silenced transformants and the wild-type strain were measured by qRT-PCR. All gene expression levels of RNAi-silenced transformants and the wild-type strain in (**a**) and (**b**) were normalized to the expression levels of β-tubulin transcripts in extracts from each sample. Bars indicate standard error. (**c**). The phenotype of RNAi-silenced transformants and the wild-type strain that grown on PDA for 10 days at 20 °C. (**d**). Comparison of the RNAi-silenced transformants and the wild-type strain for their virulence on detached leaves of tomato at 20 °C for 48 h. (**e**). Comparison of lesion diameter of the RNAi-silenced transformants and the wild-type strain. (**f**). Comparison of the hyphal growth of the RNAi-silenced transformants and the wild-type strain. P=0.05, Bars indicate standard error. The biological characterization of the RNAi-silenced transformants of other selected fungal CFPP-like associated genes is shown in Figure S1.

To confirm whether the CLP associated genes function in the development of *S. sclerotiorum*, seven of the 20 CLP associated genes in this pathway were selected to be silenced with the RNAi technique (Figure S4) because of the multi-nucleate nature of this fungus. We selected these seven genes for targeted silencing because: (i) They were all significantly up-regulated during sclerotial development or infection; (ii) Each of them was unambiguously designated as one kind of enzyme encoding gene; (iii) They were all highly expressed genes with their total number of transcripts per million clean tags (TPM) in the six libraries being above 300. These seven genes encode five different enzymes, specifically, aspartate transaminase (EC: 2.6.1.1, SS1G_03827 and SS1G_14097), phosphoenolpyruvate carboxykinase (ATP, EC: 4.1.1.49, SS1G_02281), transketolase (EC: 2.2.1.1, SS1G_10246), phosphoketolase (EC: 4.1.2.9, SS1G_06022) and fructose-bisphosphate aldolase (EC: 4.1.2.13, SS1G_06561 and SS1G_06400). For each gene, at least 30 independent transformants were obtained and confirmed through the amplification of the hygromycin resistance gene (*hph*). The abundance of the transcripts in different transformants of each gene was assessed using qRT-PCR and the significantly silenced transformants were selected for further study. The results showed that the knockdown mutants of these seven genes significantly reduced the virulence and the growth rate of *S. sclerotiorum* in varying degrees (Fig. 2 and Figure S5). Additionally, at least four genes (*SS1G_02281*, *SS1G_06022*, *SS1G_10246* and *SS1G_03827*) encoding for phosphoenolpyruvate carboxykinase (ATP), phosphoketolase, transketolase and aspartate transaminase respectively, were obviously involved in sclerotial development since their knockdown transformants showed abnormal sclerotial development or their sclerotial development was seriously delayed (Fig. 2 and Figure S5). In conclusion, many fungal CLP associated genes were found to be essential for virulence and (or) sclerotial development, suggesting that these genes gained new functions since the importance of carbon fixation in fungi is negligible when growing on organic substrates^6^.

### CFPP associated genes have undergone gene duplication, gene acquisition or loss and functional diversification in evolutionary history

**Figure 3:**
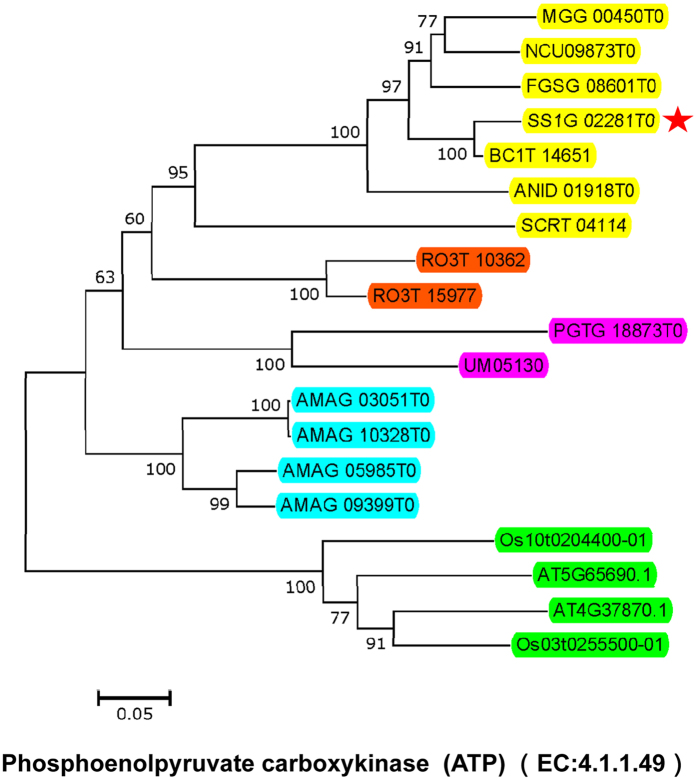
Phylogenetic analysis of CFPP and CLP associated enzyme-phosphoenolpyruvate carboxykinase (ATP) (EC: 4.1.1.49) from *S. sclerotiorum* (SS1G) and *B. cinerea* (BCIT), *A. nidulans* (ANID), *N. crassa* (NCU), *F. graminearum* (FGSG), *M. oryzae* (MGG), *S. cerevisiae* (SCRT), *P. graminis* (PGTG), *U. maydis* (UM), *R. oryzae* (RO), *A. macrogynus* (AMAG), *O. sativa* (Os) and *A. thaliana* (AT). Datasets were assembled using BLASTP (with SS1G_02281 as query sequence using a cut-off E-value of 1E-15) and the KEGG annotation. Neighbor-Joining (NJ) algorithm (1000 bootstrap replicates, with Jones-Thornton-Taylor (JTT) model support values are indicated. Locus tags in yellow, red-orange, purple, sky blue and bright green boxes for corresponding proteins indicate species of Ascomycota, Mucormycotina, Basidiomycota, Chytridiomycota and plants respectively. Red star indicates *S. sclerotiorum* protein. Scale bar corresponds to 0.05 amino acid substitutions per site. The results of phylogenetic analysis of other **CFPP and CLP** associated enzymes are presented in supplementary material Figure S6.

In order to decipher the evolutionary history and the functional relationship between the fungal CLP-related genes and the plant CFPP-associated genes, we conducted a phylogenetic analysis of the corresponding enzymes in *S. sclerotiorum*, *Botrytis cinerea*, *Aspergillus nidulans*, *Neurospora crassa*, *Fusarium graminearum*, *Magnaporthe oryzae*, *Saccharomyces cerevisiae*, *Schizosaccharomyces pombe*, *Puccinia graminis*, *Ustilago maydis*, *Rhizopus oryzae*, *Allomyces macrogynus*, *Oryza sativa* and *A. thaliana* (Fig. 3 and Figure S6). These organisms include eight Ascomycota fungi with a close evolutionary relationship to *S. sclerotiorum*, two Basidiomycota fungi, a Rhizopus (*Mucormycotina*), a chytrid (*Chytridiomycota*) and two important model plants. These fungal organisms are important to agriculture and industry, have special taxonomic status in evolution or serve as basic models for molecular and cellular biology. The results indicated that the numbers of almost all of the CFPP-associated genes in plants were more than that in fungi (Table 2, Fig. 3 and Figure S6), indicating they have undergone gene duplication events in plants which might strengthen the biological function of carbon fixation in plants. In addition, most of them were highly conserved except for fructose-bisphosphate aldolase (EC: 4.1.2.13), ribose-5-phosphate isomerase (EC: 5.3.1.6) and malate dehydrogenase (oxaloacetate-decarboxylating, NADP^+^ or decarboxylating, EC: 1.1.1.40 or EC: 1.1.1.39) which were not found in chytrid fungi. The chytrid fungi are the most primitive ancient fungi among fungi kingdom phylogenetically^20^, so it is possible that the chytrid fungi have lost these enzymes or the other fungi have obtained these enzymes through an unclear way in evolution. The phylogenetic analysis also indicated many fungal CLP associated enzymes (e.g., EC: 4.1.2.13, EC: 2.6.1.2, EC: 5.3.1.6, EC: 2.7.1.40, EC: 2.7.2.3 and EC: 4.1.1.49) have branched off from the plant CFPP-associated genes, suggesting they were likely to gain new functions in evolution. Interestingly, for each fungal CLP associated enzyme, the expanded members located in different branches with the original members, implying that the gene duplication events were followed by divergent evolutionary events in fungi. These phenomena indicated the biological functions of many CLP associated genes were in course of continuous change in evolution.

## Discussion

In this study, the “functional profile analysis” and “gene functional enrichment analysis” are complementary to each other. As expected, *S. sclerotiorum* utilizes multiple strategies synergistically for its infection, vegetative and reproductive development because many functional modules and metabolism pathways were involved in these biological processes. Many results of our functional profile analysis and gene functional enrichment analysis were coincident with previous researches, for example, our results indicated that the cell redox homeostasis associated functional modules were over-represented during the infection and sclerotial development of *S. sclerotiorum*. Actually, many researches have demonstrated the important roles of the ROS/redox “climate” or the oxidation reduction status in fungal growth and differentiation^21^,^22^, and the significant biological functions of oxidation reduction related genes during the infection and sclerotial development^23^. Another example is that our functional profile analysis suggested the fatty acid metabolic process and pigment metabolic process were intimately involved in the maturation of sclerotia (Figure S1, Table S3 and Table S4). Accordant with this, previous research has shown the maturation of sclerotia was always accompanied by the increased level of lipid peroxidation products and melanin^24^. Meanwhile, our functional profile analysis also showed the activity of lipid catabolic process associated peroxisomes were dramatically increased during the infection (Table S3), which is coincident with the crucial roles of peroxisomes in pathogenecity^25^,^26^,^27^. These suggested that the “functional profile analysis” and “gene functional enrichment analysis” approaches could be used to analyze gene expression data powerfully. Intriguingly, our “functional profile analysis” showed an incomplete fungal CLP was dramatically regulated during the development of *S. sclerotiorum*, and the subsequent functional experiments of the fungal CLP associated genes further confirmed their crucial roles during the virulence and/or sclerotial development.

As the phylogenetic analysis, some fungal CLP associated genes have undergone gene duplication events not only in plants but also in many Ascomycota fungi. For example, the phosphoketolase (EC: 4.1.2.9), aspartate transaminase (EC: 2.6.1.1), malate dehydrogenase (EC: 1.1.1.37) and fructose-bisphosphate aldolase (EC: 4.1.2.13) have two members in *S. sclerotiorum* (Table 2, Figure S6). However, for some of them, there might be no functional duplication or complementation among the two different members because they were both indispensable for the normal development of fungi. For example, both aspartate transaminase (EC: 2.6.1.1) and fructose-bisphosphate aldolase (EC: 4.1.2.13) have two members in *S. sclerotiorum*, however, all of them were necessary for the full virulence of *S. sclerotiorum*. As a special case, the phosphoketolase encoding genes are likely to come from prokaryotic organisms by horizontal gene transfer since they were found only in Ascomycota fungi and in some prokaryotes, but not in other fungi, plants and animals. However, their patchy distribution might also be caused by gene losses in multiple lineages in evolution^28^. Interestingly, our results showed that it was also involved in the virulence and sclerotial development of *S. sclerotiorum* (Figure S5). Although many fungal CLP genes were essential to the virulence of *S. sclerotiorum*, they also exist in saprophytic fungi, suggesting that the functions of these CLP associated genes have diversified in different organisms in evolution, just like Rubiscos genes in *Archaea* and *Bacillus*^29^,^30^,^31^.

In addition, our results showed that most of the components of CFPP originated from conserved enzymes involved in the basic development of organisms. Although homologs of Rubisco and PRK were not found in *S. sclerotiorum* or any other genome sequenced fungi, previous research showed that some fungi have the ability to assimilate CO_2_ and transform the inorganic carbon to organic compounds^32^. The supplementation of heterogenous PRK and Rubisco in *S. cerevisiae* also partially complement its ability of carbon fixation^9^. These results indicated the “footprint” of plant carbon fixation cycle still held the potential of carbon fixation although the functions of associated genes had diversified in evolution. These findings also indicated an enormous and untapped potential of metabolic engineering transformation of fungal strains for the carbon fixation in industrial biotechnology.

In conclusion, based on “functional profile analysis”, we found there was an incomplete CLP in fungi and the expression of many CLP associated genes was dramatically regulated during multiple developmental stages of *S. sclerotiorum*. Many CLP associated genes were experimentally confirmed to be essential to the virulence and (or) sclerotial development of S. sc*lerotiorum*. Phylogenetic analysis showed that many of them had undergone gene duplication, gene acquisition or loss and functional diversification in evolution. Our results indicated an important evolutionary linkage between the autotrophs and heterotrophs which implicated the divide between the autotrophs and heterotrophs was not so great that it could not be crossed as previously considered. Meanwhile, these findings also provided possible clues and references for the carbon fixation associated metabolic engineering.

## Methods

### *S. sclerotiorum* strains and culture conditions

In this study, wild-type *S. sclerotiorum* isolate Ep-1PNA367 was used and routinely cultured on potato dextrose agar (PDA) in an incubator at 20 °C, and stored on PDA slants at 4–6 °C. Transformants of *S. sclerotiorum* were cultured on PDA amended with 80 μg/ml hygromycin B (Calbiochem, San Diego, CA) where necessary, and stored in hygromycin-amended PDA slants.

### Nucleic acid isolation, cDNA production and quantification of gene expression by qRT-PCR amplification

Mycelia were collected and frozen in liquid nitrogen and stored at −80 °C. Genomic DNA was isolated as described by Yelton *et al.*^33^. To evaluate the dynamic expression levels of the selected genes in wild-type strain Ep-1PNA367 which were cultured on PDA from the first day to the twelfth day respectively, the cultures were collected every day to extract the total RNA. To evaluate the expression levels of the selected genes in different transformants, the transformants and the wild-type strain were cultured on PDA until the day when the expression of corresponding genes reached the highest level that is validated by the dynamic expression detection in the previous step, and then the extraction of the total RNA of these cultures, cDNA synthesis and the qRT-PCR were conducted according to Zhu *et al.*^34^. All reactions ran in triplicate by monitoring the dissociation curve to control the dimers. *S. sclerotiorum* β-tubulin gene was used as a normalizer according to Harel *et al.*^18^. After amplification, data acquisition and analysis were performed using the Bio-Rad CFX Manager Software (version 2.0).

### Digital gene expression (DGE) profiles

To collect the hyphae for RNA extraction, wild-type strain Ep-1PNA367 was grown or treated under different conditions as follows: (i) Vegetative growth stage, activating hyphal agar discs of strain Ep-1PNA367 were placed on the cellophane membrane overlaid on PDA medium and then the mycelia were collected at 12, 24, 36, 48 and 60 h; (ii) Sclerotial formation stage, the colonies growing on the cellophane membrane were further incubated at the same condition and then collected at 84, 96, 108, 120 and 132 h; (iii) Early stage of infection, fresh hyphal fragments of strain Ep-1PNA367 were overlaid on a sterilized cheese cloth which was overlaid on the leaves of *A. thaliana* ecotype Columbia-0 and incubated at 20 °C with 100% relative humility, the cheese cloth with hyphae was rolled up from the leaves at 9 h and 12 h; (iv) Sclerotial myceliogenic germination stage, sclerotia were surface sterilized with sodium hypochlorite and sowed on PDA with half body buried in a plate at 20 °C to induce myceliogenic germination, when about 50% sclerotia germinated, sclerotia were harvested; (v) Sclerotial carpogenic germination stage, sclerotia also were dried in room temperature and pretreated in a freezer (4–6 °C) for up to one month, and then were surface sterilized and sowed on wet sterilized sands with half body buried in a plate at 15 °C to induce carpogenic germination, when 50% sclerotia germinated (stipes just emerged from sclerotia), the sclerotia were harvested; (vi) Early apothecial formation stage, sclerotia were allowed to grow in the same incubator and the stipes were cut and collected just before apothecium formation. Finally, different time-point samples from the vegetative stage, sclerotial formation stage and infection stage were pooled together respectively according to equal quantities for RNA extraction and cDNA libraries construction. Illumina sequencing for DGE was performed at BGI-Shenzhen (Shenzhen, China). The DGE raw sequences were transformed into clean tags after several steps of data-processing. The clean tags were then mapped to the transcript database of the *S. sclerotiorum* (version 1) from Broad Institute (https://www.broadinstitute.org/annotation/genome/sclerotinia_sclerotiorum/MultiHome.html). The number of unambiguous clean tags for each gene was calculated before being normalized to TPM (number of transcripts per million clean tags)^35^. Rigorous algorithms were developed to identify differentially expressed genes between two samples according to Audic *et al.*^36^. In this research, P-value and FDR were manipulated to determine differentially expressed genes^37^. FDR ≤ 0.001 and the absolute value of log_2_Ratio ≥1 were used as the threshold to judge the significance of gene expression. The sequenced reads data were deposited to GEO at NCBI under accession number GSE65301.

### The genome functional annotation, the gene functional profiles and functional enrichment analysis

Predicted proteins of *A. thaliana* and *Oryzae sativa* ssp. Japonica were obtained from the Arabidopsis Information Resource (TAIR) database (http://www.arabidopsis.org/index.jsp) and the Rice Annotation Project (RAP) database (http://rapdb.dna.affrc.go.jp/) respectively. All predicted proteins of fungi were obtained from Broad Institute (https://www.broadinstitute.org/annotation/genome/sclerotinia_sclerotiorum/MultiHome.html) were annotated using Blast2GO^38^ (with the blast expect value of 1E-6) and InterProScan^39^ for the GO, IPR and KEGG annotation^16^. The identified CFPP-associated enzymes were further checked using BLASTP against the NCBI non-redundant protein database to exclude the possible false annotation. The geometric average of the expression values of all genes assigned to the same functional modules was calculated to measure their activity. To eliminate potential errors, only the functional modules in which the gene numbers >10 were calculated. In order to calculate the geometric mean of TPM values, all the values of 0 in the TPM values of DGE data were replaced by 0.001. The standard deviation of the geometric mean of the TPM values was used to reflect the change amplitude of the activity of corresponding functional modules during the six developmental stages. The functional enrichment analyses was performed by a modified Fisher’s exact test (EASE score) and three multiple testing correction techniques (Bonferroni, Benjamini and FDR) according to DAVID protocol^40^,^41^. The whole genome was used as background, and the differentially expressed genes of corresponding developmental stage (including infection, sclerotial development, myceliogenic germination, carpogenic germination and apothecium formation) compared to the vegetative growth stage were used as test gene sets. For the functional profile analysis of KEGG pathways, the variation in the geometric mean values of different stages was measured by a standard deviation test.

### RNAi vector construction and transformation

In this study, two strategies described as Yu *et al.* were adopted to construct RNAi vectors^42^. For every selected gene in this study (described as above), a 400–485 bp gene fragment was amplified with the corresponding primers from cDNA library of *S. sclerotiorum* and then (i) directly ligated into the digested pCXDPH by *Xcm* I (New England Biolabs, Beverly, MA, USA) to produce the RNAi-1 vector (Figure S4a); (ii) digested by two sets of suitable enzymes and then ligated into pCIT between P*trpC*, intron and T*trpC* respectively in opposite orientation via some intermediate vectors. Subsequently, the P*trpC*-intron-T*trpC* fragment with two target gene fragments in opposite orientation in pCIT vector was digested by *Sac* I and *Xho* I and then ligated into pCH^42^ to produce RNAi-2 vector (Figure S4b). Primers for constructing the RNAi vectors were shown in supplementary Table S7. The method of Agrobacterium-mediated transformation was used to transform *S. sclerotiorum* as described by Yu *et al.*^42^ with some modifications for Agrobacterium cultivation. The transformants from the two silencing strategies were validated by qRT-PCR and the knockdown transformants with higher silencing efficiency were selected for further study. Primers for validating the expression of transformants are shown in supplementary Table S7.

### The evaluation of biological characteristics of transformants

To evaluate the biological characteristics of silenced transformants, the transformants and the wild-type strain Ep-1PNA367 were cultivated at least three times on PDA at 20 °C using hyphal tips as inoculum. To assay growth rates, the mycelial agar discs were taken from the active colony edge and inoculated onto the center of the PDA plate, and incubated at 20 °C before hyphal growth was examined. The colony morphology of these strains was examined after being grown on PDA plate for ten days at 20 °C. To evaluate virulence, mycelial agar discs (diameter 5 mm) were inoculated onto detached tomato leaves at 20 °C for 48 h, and then the lesions induced by transformants and the wild-type strain Ep-1PNA367 were measured.

### Phylogenetic analysis of CFPP and CLP associated genes

The deduced amino acid sequences of CFPP and CLP associated genes were aligned using COBALT^43^ (http://www.ncbi.nlm.nih.gov/tools/cobalt/cobalt.cgi?link_loc=BlastHomeAd), viewed and edited in Jalview^44^. Phylogenetic trees were inferred with MEGA5^45^ by the Neighbor-Joining (NJ) algorithm that uses a matrix of pairwise distances estimated under the Jones-Thornton-Taylor (JTT) model^46^,^47^,^48^. Gaps in alignment were systematically treated as unknown characters. The reliability of internal branches was evaluated based on the bootstrap test^49^.

## Additional Information

**How to cite this article**: Lyu, X. *et al.* A “footprint” of plant carbon fixation cycle functions during the development of a heterotrophic fungus. *Sci. Rep.* **5**, 12952; doi: 10.1038/srep12952 (2015).

## Supplementary Material

Supplementary Information

Supplementary Table S1

Supplementary Table S2

Supplementary Table S3

Supplementary Table S5

Supplementary Table S6

## Figures and Tables

**Table 1:** Categorization and abundance of tags.

Summary		Sclerotial development	Vegetative growth	Myceliogenic germination	Carpogenic germination	Apothecium formation	Infection
Raw data	Total	3409189	3457606	3409426	3517668	3379115	3470463
	Distinct tags*	114837	91143	130053	113745	126988	116986
Clean tags**	Total number	3333845	3390018	3269538	3396146	3263705	3367846
	Distinct tag numbers	56749	43530	62023	66213	54974	61879
All tags mapped to genes	Total number	958990	1004950	1068344	1052053	997436	1148654
	Total % of clean tags	0.2877	0.2964	0.3268	0.3098	0.3056	0.3411
	Distinct tag numbers	17776	14034	18078	17020	17436	19178
	Distinct tags % of clean tags	0.3132	0.3224	0.2915	0.257	0.3172	0.3099
Unambiguous tags mapped to genes∆	Total number	938217	989208	1040435	1028246	977781	1128379
	Total % of clean tags	0.2814	0.2918	0.3182	0.3028	0.2996	0.335
	Distinct tag numbers	17417	13765	17701	16695	17086	18825
	Distinct tags % of clean tags	0.3069	0.3162	0.2854	0.2521	0.3108	0.3042
All tags-mapped genes	Number	8142	6901	8140	7857	8122	7866
	Percentage of reference genes	0.5607	0.4752	0.5605	0.541	0.5593	0.5417
Unambiguous tags-mapped genes	Number	7728	6525	7730	7462	7712	7463
	Percentage of reference genes	0.5322	0.4493	0.5323	0.5138	0.5311	0.5139
Mapping to genome	Total number	1612456	1711101	1770012	1869942	1972259	1789331
	Total % of clean tags	0.4837	0.5047	0.5414	0.5506	0.6043	0.5313
	Distinct tag numbers	25005	17928	25981	24212	28096	23817
	Distinct tags % of clean tags	0.4406	0.4119	0.4189	0.3657	0.5111	0.3849
Unknown tags	Total number	762399	673967	431182	474151	294010	429861
	Total % of clean tags	0.2287	0.1988	0.1319	0.1396	0.0901	0.1276
	Distinct tag numbers	13968	11568	17964	24981	9442	18884
	Distinct tags % of clean tags	0.2461	0.2657	0.2896	0.3773	0.1718	0.3052

Notes:

^*^Distinct tags indicate different kinds of tags.

^**^Clean tags indicate tags after filtering dirty tags (low-quality tags) from the raw data.

^∆^Unambiguous tags indicate the remainder of clean tags after removing tags mapped to the reference sequences from multiple genes.

**Table 2:** The fungal CLP associated genes of *S. sclerotiorum* and the corresponding homologs in *A. thaliana*.

CLP associated genes in *S. sclerotiorum*	Homologs in *A. thaliana*?	EC Code	Function
SS1G_10246	AT2G45290 (0), AT3G60750 (0)	EC: 2.2.1.1	transketolase
SS1G_03827	AT5G11520 (4E-116), AT5G19550 (1E-112), AT1G62800 (1E-107)	EC: 2.6.1.1	aspartate transaminase
SS1G_14097	AT5G11520 (1E-111),AT5G19550 (3E-111), AT1G62800 (3E-101)		
SS1G_02202	AT1G72330 (1E-119), AT1G17290 (1E-116), AT1G70580 (2E-112), AT1G23310 (3E-107)	EC: 2.6.1.2	alanine transaminase
SS1G_04568	AT4G26390 (7E-112), AT5G63680 (4E-111), AT5G08570 (6E-110), AT5G56350 (5E-108), AT3G04050 (1E-102), AT3G25960 (1E-102), AT3G55650 (3E-102), AT3G55810 (2E-88), AT5G52920 (3E-78), AT1G32440 (1E-74), AT2G36580 (5E-69), AT3G22960 (8E-67), AT3G52990 (6E-65), AT3G49160 (1E-26)	EC: 2.7.1.40	pyruvate kinase
SS1G_01105	AT1G79550 (2E-108), AT1G56190 (3E-107), AT3G12780 (1E-106),	EC: 2.7.2.3	phosphoglycerate kinase
SS1G_02281	AT4G37870 (0), AT5G65690 (4E-180)	EC: 4.1.1.49	phosphoenolpyruvate carboxykinase (ATP)
SS1G_01844	AT3G01850 (6E-60), AT1G63290 (2E-56), AT5G61410 (5E-42)	EC: 5.1.3.1	ribulose-phosphate 3-epimerase
SS1G_11433	AT3G55440 (1E-78), AT2G21170 (6E-71)	EC: 5.3.1.1	triose-phosphate isomerase
SS1G_08408	AT1G71100 (1E-19), AT2G01290 (4E-19) AT3G04790 (3E-15)	EC: 5.3.1.6	ribose-5-phosphate isomerase
SS1G_08975	AT1G53240 (4E-88), AT2G22780 (3E-84), AT3G47520 (4E-84), AT3G15020 (4E-82), AT5G09660 (3E-74)	EC: 1.1.1.37	malate dehydrogenase
SS1G_13825	AT1G53240 (2E-88), AT3G47520 (4E-85), AT2G22780 (1E-82), AT3G15020 (9E-80), AT5G09660 (7E-70)		
SS1G_11369*	AT1G43670 (9E-102), AT3G54050 (3E-77), AT5G64380 (1E-51)	EC: 3.1.3.11	fructose-bisphosphatase
	AT3G55800 (2E-21)	EC: 3.1.3.37	sedoheptulose-bisphosphatase
SS1G_07798*	AT1G79530 (2E-136), AT1G16300 (2E-134), AT3G04120 (7E-128), AT1G13440 (3E-109)	EC: 1.2.1.12	glyceraldehyde-3-phosphate dehydrogenase (phosphorylating)
	AT1G42970 (5E-77), AT1G12900 (3E-68), AT3G26650 (1E-65)	EC: 1.2.1.13	glyceraldehyde-3-phosphate dehydrogenase (NADP^+^) (phosphorylating)
SS1G_08827*	AT5G25880 (1E-103), AT1G79750 (3E-101), AT5G11670 (1E-98), AT2G19900 (1E-93),	EC: 1.1.1.40	malate dehydrogenase (oxaloacetate-decarboxylating) (NADP^+^)
	AT2G13560 (2E-86), AT4G00570 (1E-94)	EC: 1.1.1.39	malate dehydrogenase (decarboxylating)
SS1G_12079*	AT1G79750 (5E-108), AT5G25880 (1E-106), AT2G19900 (9E-106), AT5G11670 (2E-103),	EC: 1.1.1.40	malate dehydrogenase (oxaloacetate- decarboxylating) (NADP^+^)
	AT2G13560 (4E-96), AT4G00570 (2E-104)	EC: 1.1.1.39	malate dehydrogenase (decarboxylating)
SS1G_06561△		EC: 4.1.2.13	fructose-bisphosphate aldolase
SS1G_06400△			
SS1G_06022☆		EC: 4.1.2.9	phosphoketolase
SS1G_06790☆			

Notes:

^*^indicates these four proteins could not be unambiguously designated as specific enzymes with bioinformatics approaches because the similarity of the sequences of the corresponding enzymes is very high;

^∆^indicates the two proteins belong to fructose-bisphosphate aldolase class II in *S. sclerotiorum* while the corresponding homologs in *A. thaliana* belong to class I, sequence homology between the two classes is practically nonexistent;

^☆^indicates the enzyme is not exist in plants and also not necessary for the carbon fixation of plants, but it plays roles in carbon fixation in some prokaryotes;

^?^the numbers in parentheses are the E-values between CLP associated genes in *S. sclerotiorum* and their corresponding homologs in *A. thaliana*.

**Table 3:** The relative expression of CLP associated genes of *S. sclerotiorum* during different developmental stages.

Gene	Number of Transcripts Per Million clean tags (TPM)					
	Sclerotial development	Vegetative growth	Myceliogenic germination	Carpogenic germination	Apothecium	Infection
SS1G_02281*☆	119.08	2.06	314.42	66.25	34.01	312.07
SS1G_06022*☆	42.59	2.95	510.47	59.48	38.61	10.69
SS1G_02202*☆	51.59	13.57	64.23	14.72	22.67	27.61
SS1G_06400*☆	74.99	32.45	62.7	182.56	212.03	200.72
SS1G_14097*	43.79	28.61	40.68	38.87	66.8	108.97
SS1G_03827*	214.47	270.21	367.94	266.77	274.53	961.15
SS1G_10246*	316.75	204.72	554.51	167.25	308.85	530.61
SS1G_06561*	526.42	342.77	834.37	337.15	449.8	690.95
SS1G_11369*	119.68	83.48	162.71	112.48	104.18	426.98
SS1G_08975☆	14.4	5.6	10.7	3.83	7.97	8.31
SS1G_12079☆	18	5.01	142.22	14.13	31.56	4.75
SS1G_01844△	47.99	60.47	42.82	35.63	20.53	25.83
SS1G_04568△	542.62	387.9	198.19	211.42	187.52	179.94
SS1G_07798△	33.89	41	19.27	14.13	6.43	16.92
SS1G_08408	0	1.18	1.84	2.65	0	5.05
SS1G_11433	301.45	304.42	449.3	327.43	199.16	441.23
SS1G_01105	1.5	1.18	0.92	1.47	0	1.78
SS1G_06790	4.5	0.59	3.98	4.42	1.23	2.08
SS1G_08827	0	0	0	0.59	0.61	0.59
SS1G_13825	0.6	3.83	2.45	2.06	3.37	4.75

Notes:

^*^ and ^∆^indicates the significantly up-regulated and down-regulated genes respectively during infection by DGE data analysis;

^☆^indicates the significantly up-regulated genes during sclerotial development by DGE data analysis.
