# Supplementary material for: A “footprint” of plant carbon fixation cycle functions during the development of a heterotrophic fungus
Source: Sci Rep. 2015 Aug 11;5:12952. doi: 10.1038/srep12952 (PMC4642529; doi:10.1038/srep12952)
Supplement: Supplementary Information [file srep12952-s1.pdf]

A “footprint” of plant carbon fixation cycle functions during the development of a heterotrophic fungus

Xueliang Lyu[1,2], Cuicui Shen[1,2], JiataoXie[2], Yanping Fu[2], Daohong Jiang[1,2], Zijin Hu[1,2], Lihua Tang[1,2], Liguang Tang[1,2], Feng Ding[1,2], Kunfei Li[1,2], Song Wu[1,2], Yanping Hu[1,2], Lilian Luo[1,2], Yuanhao Li[1,2], Qihua Wang[1,2], Guoqing Li[1,2], Jiasen Cheng[1,2]*

1 State Key Laboratory of Agricultural Microbiology, Huazhong Agricultural University, Wuhan 430070, Hubei Province, China

2 The Provincial Key Lab of Plant Pathology of Hubei Province, College of Plant Science and Technology, Huazhong Agricultural University, Wuhan 430070, Hubei Province, China

* Corresponding author:

Jiasen Cheng, Tel: +86-27-87280487, E-mail: jiasencheng@mail.hzau.edu.cn

Supplementary information

Figure S1. The functional profile analysis. The relative activity of partial GO terms of biological processes (a), molecular functions (b) and cell components (c) during the six developmental stages of *S. sclerotiorum*. More details and the functional profile analysis of KEGG pathways see supplementary Table S3 and Table S4 respectively.

Figure S2. The gene functional enrichment analysis. Partial enriched GO and InterPro functional categories during the infection (a), sclerotial development (b), myceliogenic germination (c), carpogenic germination (d) and apothecium formation (e). The functional categories of p-value < 0.05 and fold enrichment > 2 were showed. More details and the enriched KEGG pathways see supplementary Table S5 and Table S6 respectively.

Figure S3. QRT-PCR validation of the DGE data. The TPM values in the DGE data and the expression change folds from qRT-PCR analysis were used to compare. Relative quantitation was performed to measure the changes in target gene expression in the six cDNA libraries. The quantity of target gene cDNA measured by qRT-PCR was normalized to that of β-tubulin cDNA within each reaction. The relative abundance of target gene cDNA from the vegetative stage was arbitrarily assigned a value of 1.0. Standard error of qRT-PCR is shown. Pearson's correlation coefficient (R value) was used to measure the consistency of the qRT-PCR and DGE data.

Figure S4. Construction of RNAi vectors of target genes. (a). A fragment of each selected gene was amplified with the corresponding primers and inserted between the *N. crassa trpC* promoter P*trpC* and *A. nidulans gpd* promoter P*gpd*. (b) A fragment of each selected gene was amplified with the corresponding primers and digested by two sets of appropriate enzymes respectively to produce two fragments with different sticky ends, and then the two fragments were inserted between the *N. crassa trpC*

promoter P*trpC*, an intron from *G. zeae*, and the *N. crassa trpC* terminator T*trpC*, respectively. The two fragments were in a reverse orientation.

Figure S5. Biological characterization of the RNAi-silenced transformants of fungal CLP associated genes including *SS1G_06022, SS1G_10246, SS1G_03827, SS1G_14097, SS1G_06561* and *SS1G_06400*. For each selected gene, (a). qRT-PCR analysis of gene transcripts from the first day to the twelfth day, a value of 1 was assigned to the abundance of cDNA from the first day's mycelia mass. (b). Relative expression levels of CLP associated genes in the RNAi-silenced transformants and the wild-type strain determined with qRT-PCR, a value of 1 was assigned to the abundance of cDNA from the wild-type strain. All gene expression levels of the RNAi-silenced transformants and the wild-type strain in (a) and (b) were normalized to the expression levels of β-tubulin transcripts in extracts from each sample. (c). Phenotype of the RNAi-silenced transformants and the wild-type strain that grown on PDA for ten days at 20 °C. (d). Comparison of the RNAi-silenced transformants and the wild-type strain for their virulence on detached leaves of tomato at 20 °C for 48 h. (e). Comparison of lesion diameter of the RNAi-silenced transformants and the wild-type strain. (f). Comparison of hypha growth rate of the RNAi-silenced transformants and the wild-type strain. P=0.05, Bars indicate standard error.

Figure S6. Phylogenetic analysis of other CFPP and CLP associated enzymes of *S. sclerotiorum* (SS1G) and other selected organisms. These organisms are: *B. cinerea* (BCIT), *A. nidulans* (ANID), *N. crassa* (NCU), *F. graminearum* (FGSG), *M. oryzae* (MGG), *S. cerevisiae* (SCRT), *S. pombe* (SP), *P. graminis* (PGTG), *U. maydis* (UM), *R. oryzae* (RO), *A. macrogynus* (AMAG), *O. sativa* (Os) and *A. thaliana* (AT). These enzymes are: (a). phosphoketolase (EC: 4.1.2.9); (b). aspartate transaminase (EC: 2.6.1.1); (c). triose-phosphate isomerase (EC: 5.3.1.1); (d). transketolase (EC: 2.2.1.1);

(e). fructose-bisphosphatase (EC: 3.1.3.11); (f). glyceraldehyde-3-phosphate dehydrogenase (phosphorylating and $NADP^+$, phosphorylating, EC: 1.2.1.12 and EC: 1.2.1.13); (g). alanine transaminase (EC: 2.6.1.2); (h). ribulose-phosphate 3-epimerase (EC: 5.1.3.1); (i). pyruvate kinase (EC: 2.7.1.40); (j). fructose-bisphosphate aldolase (EC: 4.1.2.13); (k). malate dehydrogenase (EC: 1.1.1.37); (l). ribose-5-phosphate isomerase (EC: 5.3.1.6); (m). phosphoglycerate kinase (EC: 2.7.2.3); and (n). malate dehydrogenase (oxaloacetate-decarboxylating, $NADP^+$ and decarboxylating, EC: 1.1.1.40 and EC: 1.1.1.39); Datasets were assembled by using BLASTP (with query sequences SS1G_06022, SS1G_06790, SS1G_10246, SS1G_03827, SS1G_14097, SS1G_06561, SS1G_06400, SS1G_02202, SS1G_11369, SS1G_01844, SS1G_08408, SS1G_04568, SS1G_13825, SS1G_08975, SS1G_11433, SS1G_01105, SS1G_08827, SS1G_12079 and SS1G_07798 with a cut-off E-value of 1E-15) and the KEGG annotation. Neighbor-Joining (NJ) algorithm (1000 bootstrap replicates, with Jones-Thornton-Taylor (JTT) model) support values are indicated. Locus tags (containing version information in Broad Institute) in yellow, red-orange, purple, sky blue and bright green boxes for corresponding proteins indicate species of Ascomycota, Mucormycotina, Basidiomycota, Chytridiomycota and plant respectively. Red stars indicate *S. sclerotiorum* proteins. Scale bars correspond to amino acid substitutions per site.

Table S1. The expression profiles of *S. sclerotiorum*. TPM, normalized clean tag number.

Table S2. The genome annotations of *S. sclerotiorum* by IPR, GO and KEGG respectively.

Table S3. The GO functional profile analysis. Numbers represent the relative activity of all GO categories including biological processes, molecular functions and

cell components during different developmental stages. The relative activity is measured by the geometric average of the expression values of all genes in corresponding GO categories. Note: * indicates the number of proteins assigned to corresponding pathway; ** indicates the standard deviation of the geometric mean of the TPM values of the six representative cDNA libraries.

Table S4. The KEGG functional profile analysis. Numbers represent the relative activity of all KEGG metabolic pathways during different developmental stages. The relative activity is measured by the geometric average of the expression values of all genes in corresponding KEGG metabolic pathways. Note: * indicates the number of enzymes assigned to corresponding pathway; ** indicates the standard deviation of the geometric mean of the TPM values of the six representative cDNA libraries.

Table S5. The GO gene functional enrichment analysis. Tables showed the over-represented GO and InterPro functional categories during different developmental stages compared to the vegetative growth stage. The enriched GO and InterPro terms of p-value (EASE score) < 0.1 in the modified Fisher's exact test were listed.

Table S6. The KEGG metabolic pathway enrichment analysis. Tables showed the over-represented KEGG metabolic pathways during different developmental stages compared to the vegetative growth stage.

Table S7. Primers used in this study.

Figure S1

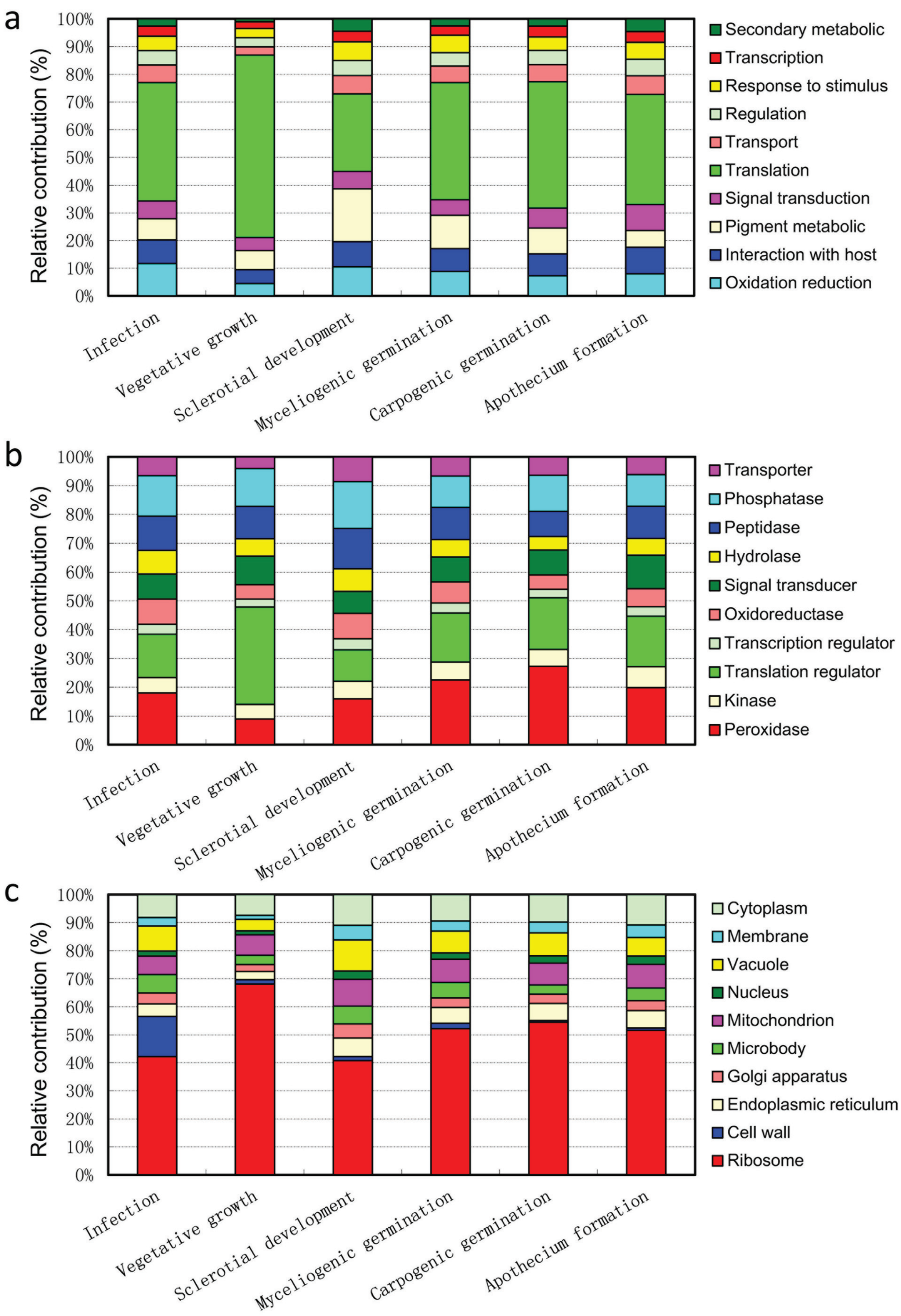

a
b
c
Relative contribution (%)
0%
10%
20%
30%
40%
50%
60%
70%
80%
90%
100%
Infection
Vegetative growth
Sclerotial development
Myceliogenic germination
Carpogenic germination
Apothecium formation
Secondary metabolic
Transcription
Response to stimulus
Regulation
Transport
Translation
Signal transduction
Pigment metabolic
Interaction with host
Oxidation reduction
Transporter
Phosphatase
Peptidase
Hydrolase
Signal transducer
Oxidoreductase
Transcription regulator
Translation regulator
Kinase
Peroxidase
Cytoplasm
Membrane
Vacuole
Nucleus
Mitochondrion
Microbody
Golgi apparatus
Endoplasmic reticulum
Cell wall
Ribosome

Figure S2

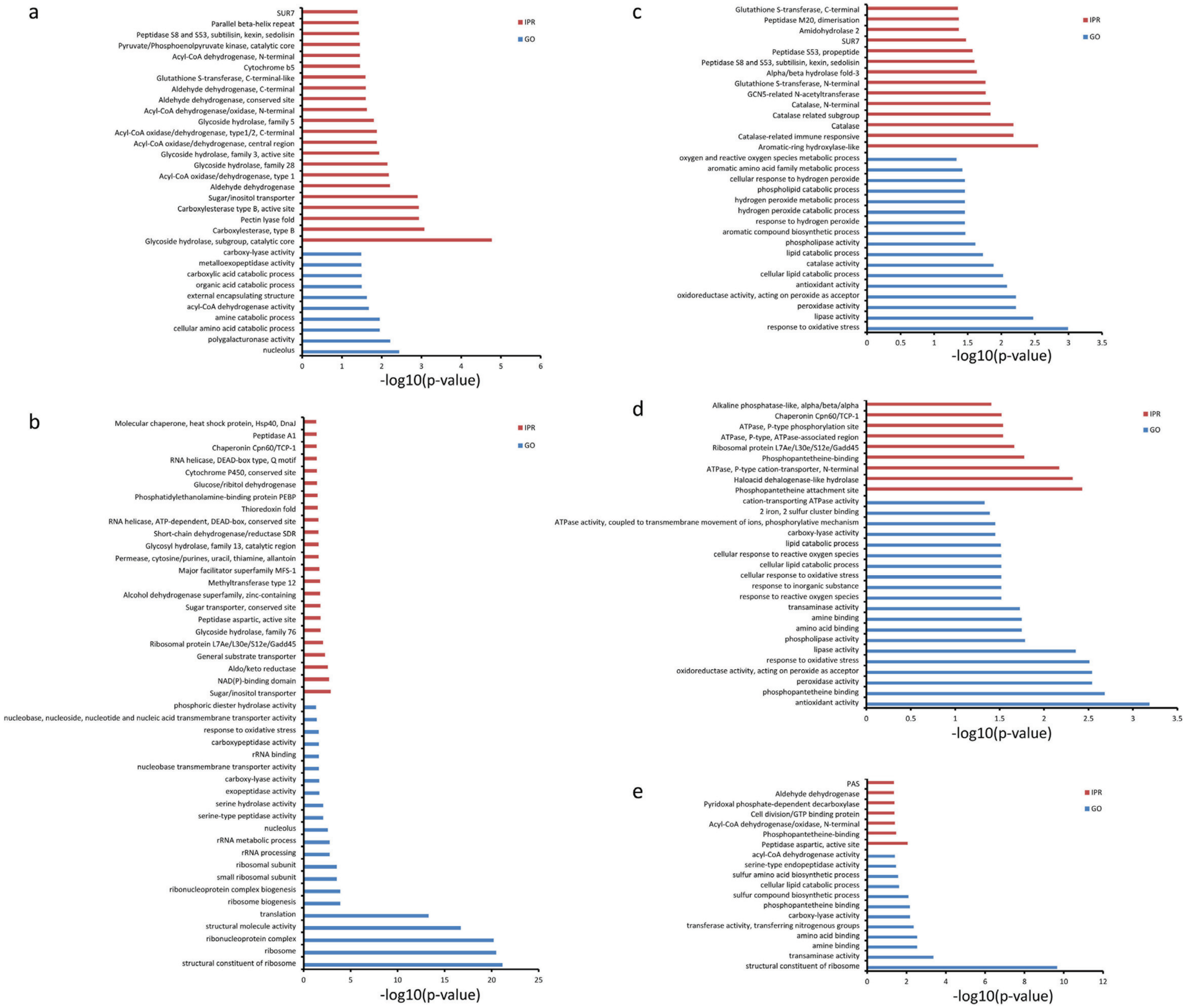

a
SUR7
Parallel beta-helix repeat
Peptidase S8 and S53, subtilisin, kexin, sedolisin
Pyruvate/Phosphoenolpyruvate kinase, catalytic core
Acyl-CoA dehydrogenase, N-terminal
Cytochrome b5
Glutathione S-transferase, C-terminal-like
Aldehyde dehydrogenase, C-terminal
Aldehyde dehydrogenase, conserved site
Acyl-CoA dehydrogenase/oxidase, N-terminal
Glycoside hydrolase, family 5
Acyl-CoA oxidase/dehydrogenase, type1/2, C-terminal
Acyl-CoA oxidase/dehydrogenase, central region
Glycoside hydrolase, family 3, active site
Glycoside hydrolase, family 28
Acyl-CoA oxidase/dehydrogenase, type 1
Aldehyde dehydrogenase
Sugar/inositol transporter
Carboxylesterase type B, active site
Pectin lyase fold
Carboxylesterase, type B
Glycoside hydrolase, subgroup, catalytic core
carboxy-lyase activity
metalloexopeptidase activity
carboxylic acid catabolic process
organic acid catabolic process
external encapsulating structure
acyl-CoA dehydrogenase activity
amine catabolic process
cellular amino acid catabolic process
polygalacturonase activity
nucleolus
IPR
GO
0
1
2
3
4
5
6
-log10(p-value)
b
Molecular chaperone, heat shock protein, Hsp40, DnaJ
Peptidase A1
Chaperonin Cpn60/TCP-1
RNA helicase, DEAD-box type, Q motif
Cytochrome P450, conserved site
Glucose/ribitol dehydrogenase
Phosphatidylethanolamine-binding protein PEBP
Thioredoxin fold
RNA helicase, ATP-dependent, DEAD-box, conserved site
Short-chain dehydrogenase/reductase SDR
Glycosyl hydrolase, family 13, catalytic region
Permease, cytosine/purines, uracil, thiamine, allantoin
Major facilitator superfamily MFS-1
Methyltransferase type 12
Alcohol dehydrogenase superfamily, zinc-containing
Sugar transporter, conserved site
Peptidase aspartic, active site
Glycoside hydrolase, family 76
Ribosomal protein L7Ae/L30e/S12e/Gadd45
General substrate transporter
Aldo/keto reductase
NAD(P)-binding domain
Sugar/inositol transporter
phosphoric diester hydrolase activity
nucleobase, nucleoside, nucleotide and nucleic acid transmembrane transporter activity
response to oxidative stress
carboxypeptidase activity
rRNA binding
nucleobase transmembrane transporter activity
carboxy-lyase activity
exopeptidase activity
serine hydrolase activity
serine-type peptidase activity
nucleolus
rRNA metabolic process
rRNA processing
ribosomal subunit
small ribosomal subunit
ribonucleoprotein complex biogenesis
ribosome biogenesis
translation
structural molecule activity
ribonucleoprotein complex
ribosome
structural constituent of ribosome
IPR
GO
0
5
10
15
20
25
-log10(p-value)
c
Glutathione S-transferase, C-terminal
Peptidase M20, dimerisation
Amidohydrolase 2
SUR7
Peptidase S53, propeptide
Peptidase S8 and S53, subtilisin, kexin, sedolisin
Alpha/beta hydrolase fold-3
Glutathione S-transferase, N-terminal
GCN5-related N-acetyltransferase
Catalase, N-terminal
Catalase related subgroup
Catalase
Catalase-related immune responsive
Aromatic-ring hydroxylase-like
oxygen and reactive oxygen species metabolic process
aromatic amino acid family metabolic process
cellular response to hydrogen peroxide
phospholipid catabolic process
hydrogen peroxide metabolic process
hydrogen peroxide catabolic process
response to hydrogen peroxide
aromatic compound biosynthetic process
phospholipase activity
lipid catabolic process
catalase activity
cellular lipid catabolic process
antioxidant activity
oxidoreductase activity, acting on peroxide as acceptor
peroxidase activity
lipase activity
response to oxidative stress
IPR
GO
0
0.5
1
1.5
2
2.5
3
3.5
-log10(p-value)
d
Alkaline phosphatase-like, alpha/beta/alpha
Chaperonin Cpn60/TCP-1
ATPase, P-type phosphorylation site
ATPase, P-type, ATPase-associated region
Ribosomal protein L7Ae/L30e/S12e/Gadd45
Phosphopantetheine-binding
ATPase, P-type cation-transporter, N-terminal
Haloacid dehalogenase-like hydrolase
Phosphopantetheine attachment site
cation-transporting ATPase activity
2 iron, 2 sulfur cluster binding
ATPase activity, coupled to transmembrane movement of ions, phosphorylative mechanism
carboxy-lyase activity
lipid catabolic process
cellular response to reactive oxygen species
cellular lipid catabolic process
cellular response to oxidative stress
response to inorganic substance
response to reactive oxygen species
transaminase activity
amine binding
amino acid binding
phospholipase activity
lipase activity
response to oxidative stress
oxidoreductase activity, acting on peroxide as acceptor
peroxidase activity
phosphopantetheine binding
antioxidant activity
IPR
GO
0
0.5
1
1.5
2
2.5
3
3.5
-log10(p-value)
e
PAS
Aldehyde dehydrogenase
Pyridoxal phosphate-dependent decarboxylase
Cell division/GTP binding protein
Acyl-CoA dehydrogenase/oxidase, N-terminal
Phosphopantetheine-binding
Peptidase aspartic, active site
acyl-CoA dehydrogenase activity
serine-type endopeptidase activity
sulfur amino acid biosynthetic process
cellular lipid catabolic process
sulfur compound biosynthetic process
phosphopantetheine binding
carboxy-lyase activity
transferase activity, transferring nitrogenous groups
amino acid binding
amine binding
transaminase activity
structural constituent of ribosome
IPR
GO
0
2
4
6
8
10
12
-log10(p-value)

Figure S3

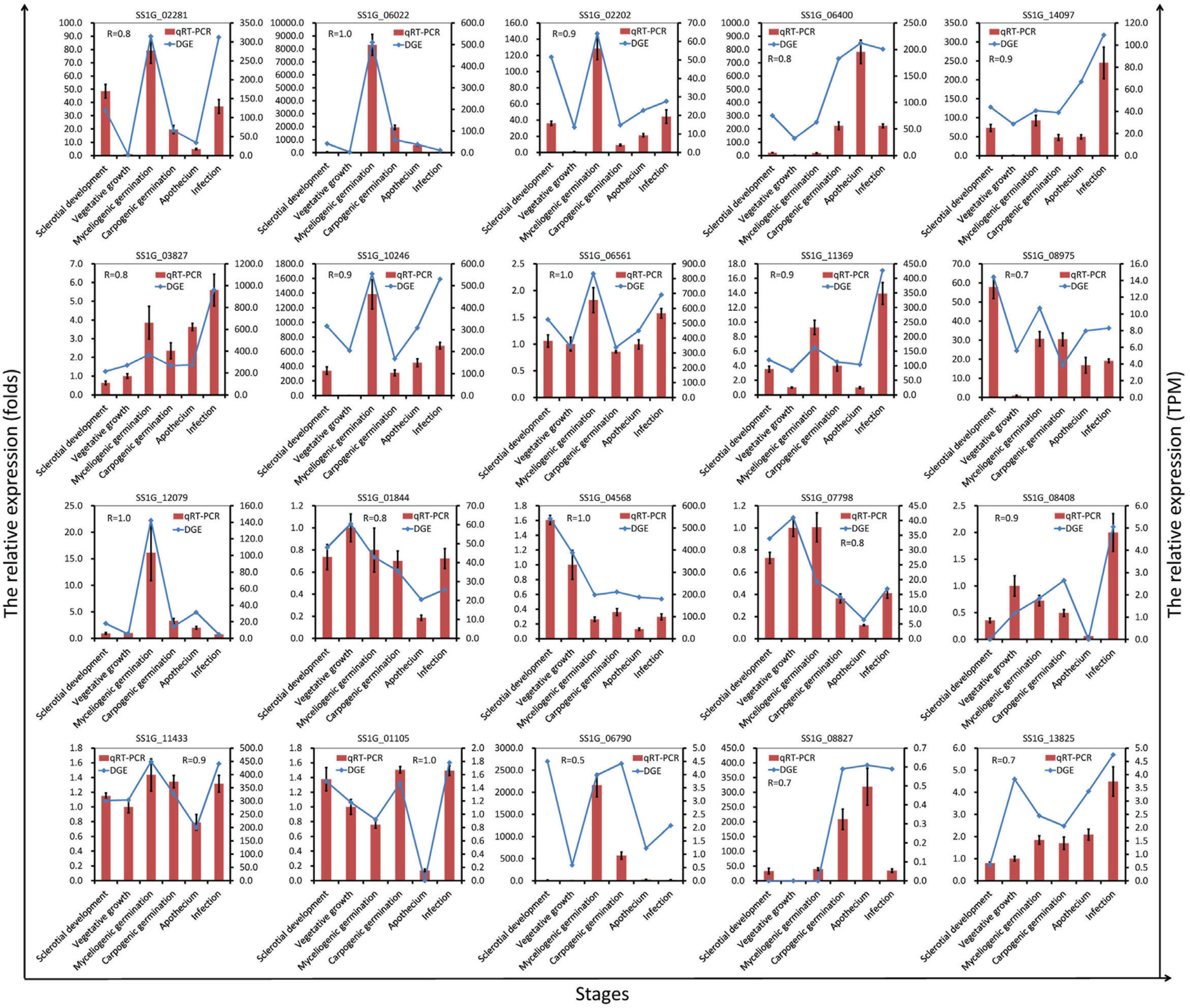

The relative expression (folds)
The relative expression (TPM)
Stages
qRT-PCR
DGE
Sclerotial development
Vegetative growth
Myceliogenic germination
Carpogenic germination
Apothecium
Infection
SS1G_02281 R=0.8
SS1G_06022 R=1.0
SS1G_02202 R=0.9
SS1G_06400 R=0.8
SS1G_14097 R=0.9
SS1G_03827 R=0.8
SS1G_10246 R=0.9
SS1G_06561 R=1.0
SS1G_11369 R=0.9
SS1G_08975 R=0.7
SS1G_12079 R=1.0
SS1G_01844 R=0.8
SS1G_04568 R=1.0
SS1G_07798 R=0.8
SS1G_08408 R=0.9
SS1G_11433 R=0.9
SS1G_01105 R=1.0
SS1G_06790 R=0.5
SS1G_08827 R=0.7
SS1G_13825 R=0.7

Figure S4

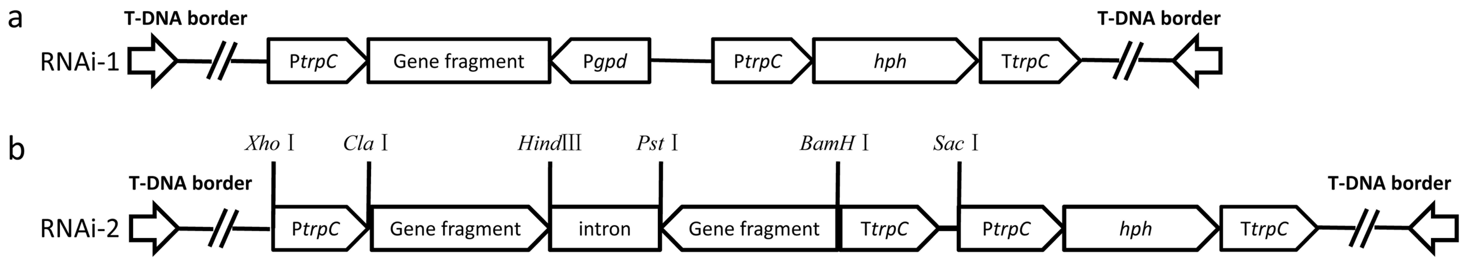
a
T-DNA border
RNAi-1
PtrpC
Gene fragment
Pgpd
PtrpC
hph
TtrpC
T-DNA border
b
Xho I
Cla I
HindIII
Pst I
BamH I
Sac I
T-DNA border
RNAi-2
PtrpC
Gene fragment
intron
Gene fragment
TtrpC
PtrpC
hph
TtrpC
T-DNA border

Figure S5

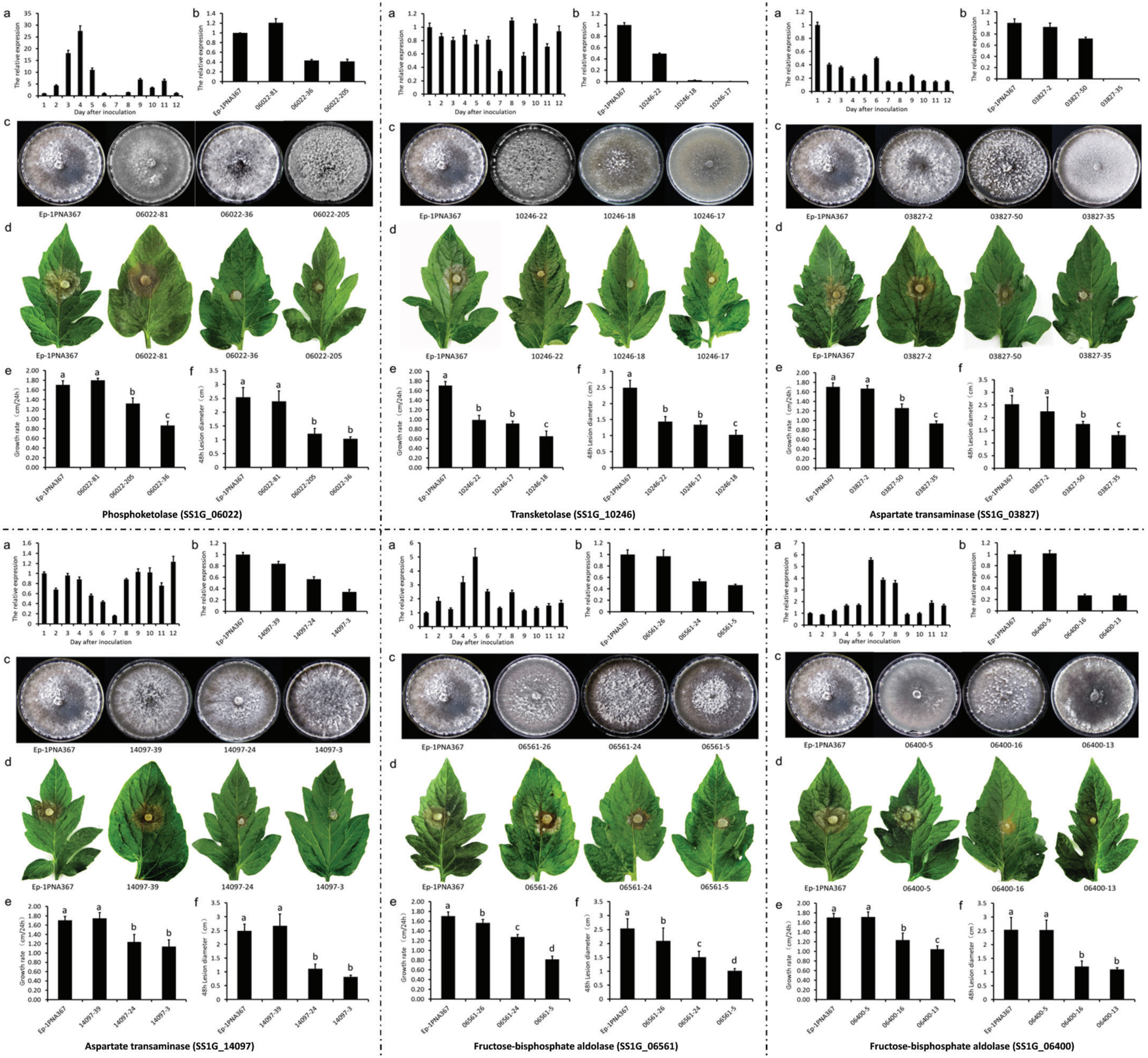

Phosphoketolase (SS1G_06022)
Ep-1PNA367
06022-81
06022-36
06022-205
Transketolase (SS1G_10246)
10246-22
10246-18
10246-17
Aspartate transaminase (SS1G_03827)
03827-2
03827-50
03827-35
Aspartate transaminase (SS1G_14097)
14097-39
14097-24
14097-3
Fructose-bisphosphate aldolase (SS1G_06561)
06561-26
06561-24
06561-5
Fructose-bisphosphate aldolase (SS1G_06400)
06400-5
06400-16
06400-13
The relative expression
Day after inoculation
Growth rate (cm/24h)
48h Lesion diameter (cm)

Figure S6

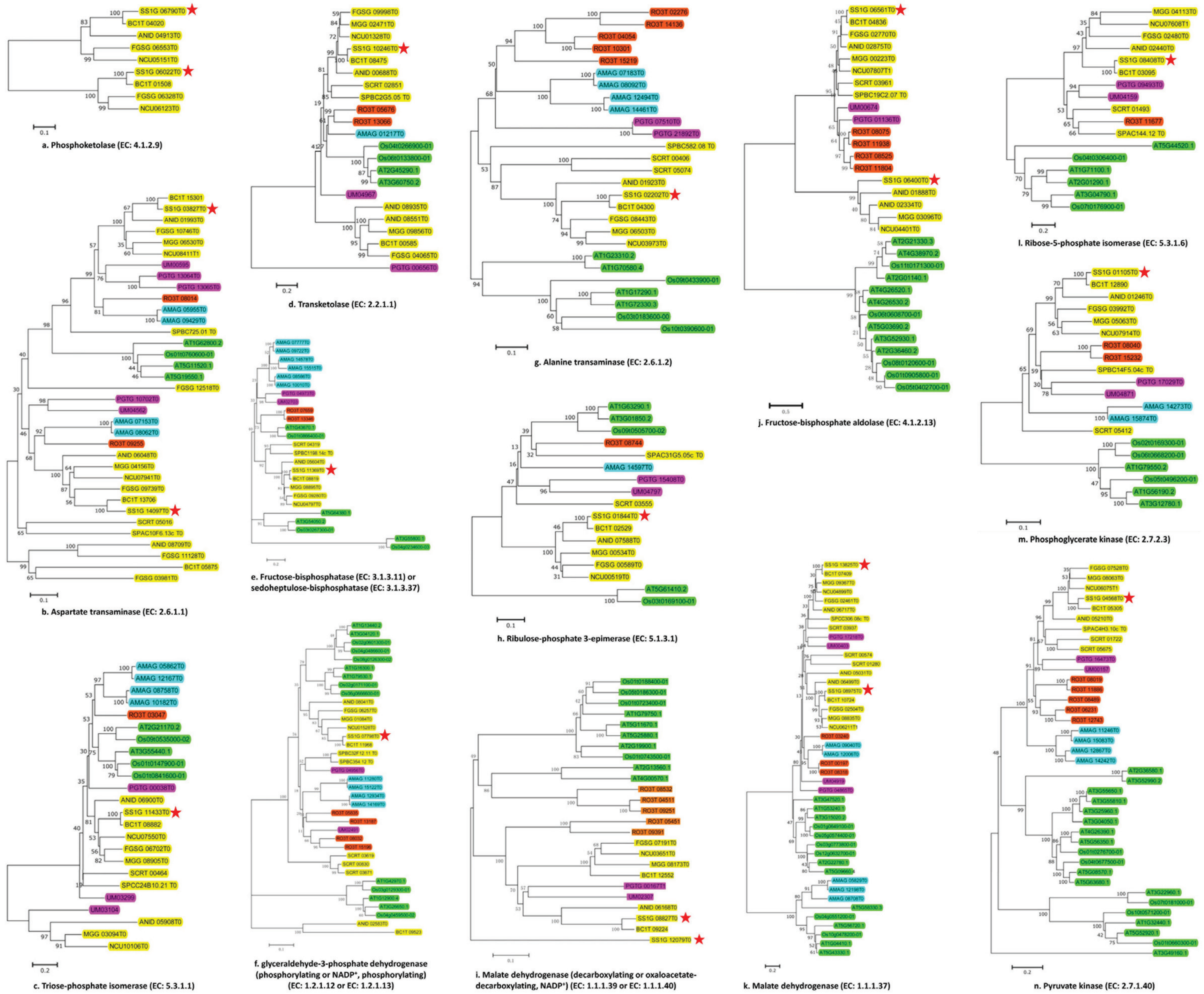

a. Phosphoketolase (EC: 4.1.2.9)

b. Aspartate transaminase (EC: 2.6.1.1)

c. Triose-phosphate isomerase (EC: 5.3.1.1)

d. Transketolase (EC: 2.2.1.1)

e. Fructose-bisphosphatase (EC: 3.1.3.11) or sedoheptulose-bisphosphatase (EC: 3.1.3.37)

f. glyceraldehyde-3-phosphate dehydrogenase (phosphorylating or NADP⁺, phosphorylating) (EC: 1.2.1.12 or EC: 1.2.1.13)

g. Alanine transaminase (EC: 2.6.1.2)

h. Ribulose-phosphate 3-epimerase (EC: 5.1.3.1)

i. Malate dehydrogenase (decarboxylating or oxaloacetate-decarboxylating, NADP⁺) (EC: 1.1.1.39 or EC: 1.1.1.40)

j. Fructose-bisphosphate aldolase (EC: 4.1.2.13)

k. Malate dehydrogenase (EC: 1.1.1.37)

l. Ribose-5-phosphate isomerase (EC: 5.3.1.6)

m. Phosphoglycerate kinase (EC: 2.7.2.3)

n. Pyruvate kinase (EC: 2.7.1.40)

Table S4. The KEGG functional profile analysis

KEGG Pathways	Geometric mean of TPM values ☆						Protein numbers *	Mean	SD **
	Sclerotial development	Vegetative growth	Myceliogenic germination	Carpogenic germination	Apothecium formation	Infection			
Fatty acid metabolism	24.19	11.46	38.16	33.97	32.11	72.59	18	35.41	20.50
Glycolysis / Gluconeogenesis	51.45	31.05	63.86	40.86	38.91	69.86	26	49.33	15.18
Tyrosine metabolism	25.24	8.90	31.62	12.53	17.65	39.85	11	22.63	11.83
beta-Alanine metabolism	18.33	10.05	28.01	24.07	23.27	44.98	17	24.78	11.66
Photosynthesis	75.32	80.67	88.95	65.92	99.27	83.82	10	82.32	11.44
**Carbon fixation in photosynthetic organisms**	**15.87**	**11.88**	**32.09**	**26.42**	**12.19**	**36.89**	**20**	**22.56**	**10.75**
Galactose metabolism	9.07	5.69	10.20	6.72	10.19	33.63	17	12.58	10.47
Propanoate metabolism	27.98	20.63	31.29	21.48	30.91	48.21	23	30.08	9.98
Valine, leucine and isoleucine degradation	15.45	10.47	15.57	12.73	15.42	32.66	24	17.05	7.91
Reductive carboxylate cycle (CO2 fixation)	21.63	10.88	28.35	9.59	20.75	13.22	14	17.40	7.35
Citrate cycle (TCA cycle)	16.35	12.65	27.05	8.62	17.44	21.53	28	17.27	6.49
Pyruvate metabolism	14.78	11.23	27.36	12.73	21.84	22.82	25	18.46	6.45
Glyoxylate and dicarboxylate metabolism	8.56	12.37	19.29	7.43	9.43	22.84	14	13.32	6.32
Cyanoamino acid metabolism	2.03	0.99	2.50	1.89	2.92	16.88	10	4.53	6.08
Oxidative phosphorylation	20.30	20.81	33.47	16.68	21.64	26.37	42	23.21	5.91
Histidine metabolism	23.96	19.16	26.31	9.43	19.46	19.57	13	19.65	5.79
Pantothenate and CoA biosynthesis	7.64	13.39	12.04	6.14	11.66	22.19	13	12.18	5.64
Nitrogen metabolism	7.04	3.60	6.09	5.34	3.59	17.48	22	7.19	5.22
Biosynthesis of alkaloids derived from ornithine, lysine and nicotinic acid	24.22	23.93	23.14	11.97	16.98	22.50	51	20.46	4.93
Glycerolipid metabolism	5.77	1.65	8.04	4.34	6.04	15.57	21	6.90	4.75
Porphyrin and chlorophyll metabolism	4.10	2.91	15.05	3.96	6.04	3.14	12	5.87	4.63
Drug metabolism - other enzymes	1.49	2.27	4.92	3.47	1.07	13.03	11	4.38	4.47
Glycine, serine and threonine metabolism	12.49	13.04	20.13	9.92	11.38	19.91	25	14.48	4.42

KEGG Pathways	Geometric mean of TPM values ☆						Protein numbers *	Mean	SD **
	Sclerotial development	Vegetative growth	Myceliogenic germination	Carpogenic germination	Apothecium formation	Infection			
Fructose and mannose metabolism	15.85	4.92	16.01	9.18	10.84	9.20	19	11.00	4.29
Sulfur metabolism	6.41	11.03	13.85	12.05	4.19	5.21	13	8.79	4.02
Methane metabolism	11.45	3.07	12.08	12.37	6.74	6.47	26	8.70	3.82
Selenoamino acid metabolism	7.83	10.53	8.36	8.18	5.15	16.22	14	9.38	3.76
Biosynthesis of alkaloids derived from terpenoid and polyketide	14.36	16.96	17.59	7.17	15.27	14.53	51	14.31	3.73
Starch and sucrose metabolism	3.86	1.20	2.81	1.21	0.98	10.19	48	3.38	3.53
Amino sugar and nucleotide sugar metabolism	12.36	2.55	8.39	7.36	9.94	11.13	38	8.63	3.48
Tryptophan metabolism	8.18	1.67	12.35	8.58	6.88	7.80	23	7.58	3.45
One carbon pool by folate	7.06	13.24	6.65	6.64	4.23	3.99	13	6.97	3.34
Biosynthesis of alkaloids derived from shikimate pathway	13.21	15.08	17.78	9.42	11.21	17.28	66	14.00	3.33
Valine, leucine and isoleucine biosynthesis	14.73	16.17	19.12	11.34	16.56	20.65	19	16.43	3.28
Butanoate metabolism	14.74	6.61	11.60	6.77	8.04	8.86	17	9.44	3.17
Phenylalanine metabolism	11.32	4.46	9.15	8.24	7.78	13.66	18	9.10	3.15
Biosynthesis of terpenoids and steroids	11.65	13.79	14.30	5.93	13.46	11.68	54	11.80	3.08
Biosynthesis of alkaloids derived from histidine and purine	12.26	17.01	19.03	10.77	14.15	15.68	70	14.82	3.05
Pentose phosphate pathway	10.39	6.79	14.66	13.36	11.76	9.94	23	11.15	2.78
Pentose and glucuronateinterconversions	1.33	0.39	0.88	0.37	0.43	7.39	25	1.80	2.77
Lysine degradation	4.02	4.35	7.07	3.16	10.14	7.30	15	6.01	2.63
Glutathione metabolism	6.58	3.62	7.01	7.92	10.78	8.66	19	7.43	2.38
Alanine, aspartate and glutamate metabolism	4.37	6.16	4.47	7.24	3.29	8.69	28	5.70	2.03
N-Glycan biosynthesis	4.74	4.03	2.41	1.64	6.49	6.26	18	4.26	1.98
Biosynthesis of phenylpropanoids	10.08	7.18	11.55	7.40	8.76	10.56	82	9.25	1.77
gamma-Hexachlorocyclohexane degradation	5.42	1.26	2.13	3.08	2.09	4.22	15	3.03	1.55
Aminoacyl-tRNA biosynthesis	6.15	7.81	6.48	3.78	4.11	5.24	38	5.60	1.52
Phenylalanine, tyrosine and tryptophan biosynthesis	4.71	6.02	8.04	5.12	5.17	7.89	30	6.16	1.46

KEGG Pathways	Geometric mean of TPM values ☆						Protein numbers *	Mean	SD **
	Sclerotial development	Vegetative growth	Myceliogenic germination	Carpogenic germination	Apothecium formation	Infection			
Biosynthesis of plant hormones	8.07	9.35	10.89	6.50	8.88	8.86	115	8.76	1.45
Arginine and proline metabolism	7.08	4.02	4.19	3.55	3.03	4.77	39	4.44	1.42
Inositol phosphate metabolism	4.79	4.31	6.49	4.66	5.93	8.00	10	5.70	1.40
Thiamine metabolism	6.19	2.70	3.87	3.92	3.39	2.65	49	3.79	1.30
Glycerophospholipid metabolism	5.12	1.61	3.75	4.41	3.88	2.94	16	3.62	1.22
Cysteine and methionine metabolism	7.01	8.74	10.32	8.59	8.71	7.31	28	8.45	1.19
Axon guidance	3.30	0.83	3.12	1.35	1.19	2.61	15	2.07	1.07
Pyrimidine metabolism	1.90	3.52	2.16	2.42	1.10	1.23	54	2.05	0.89
Riboflavin metabolism	3.97	2.01	2.14	1.84	1.96	2.42	16	2.39	0.80
Purine metabolism	3.00	2.89	2.88	2.98	1.46	1.40	126	2.43	0.78
Phosphatidylinositol signaling system	1.58	0.59	2.26	0.83	1.32	1.52	10	1.35	0.59

Table S7. Primers used in this study

Genes	Primer name	Forward primer (5’-3’)	Reverse primer (5’-3’)	Use
SS1G_02281	RNAi1-02281 F/R	GCATCGGTCTATCAGCGGAGAAGGAG	CCATCCAGTGTTCAACAACCAAGCATTAGC	For constructing RNAi-1 vector
SS1G_03827	RNAi1-03827 F/R	AATGGAAGGAGATCAGCGAAGTTGTCAAGG	GGCTAGTGATGTGAGACCAATCGTGCTT	For constructing RNAi-1 vector
SS1G_04568	RNAi1-04568 F/R	GGATCAGAGATCAACATTACAACCGATGACAAGT	AATCACCACGAGCAACCATGACACCAT	For constructing RNAi-1 vector
SS1G_06022	RNAi1-06022 F/R	CGTCGTCGCCAACAAGAGTCCTGAAG	ATGGGTAGGAGTGGAAATTAAAGATGATGGGTTTG	For constructing RNAi-1 vector
SS1G_06400	RNAi1-06400 F/R	AGCAGAAGACGGTGGTTATGGTGTTGT	TCAGGAGTCGTGAGTAACCCTTCCAAATC	For constructing RNAi-1 vector
SS1G_06561	RNAi1-06561 F/R	TGACAACAACTCCCTCTACACTCAACCA	TTTCTCTCCCTCACGAACCCAGACAC	For constructing RNAi-1 vector
SS1G_10246	RNAi1-10246 F/R	AAGATTCACGAGGCTATTCCAGAACTTGTTG	GAAGGAGTGTGCTTGGAGGTCAATGC	For constructing RNAi-1 vector
SS1G_11433	RNAi1-11433 F/R	TTCGTTGGAGGTAACTTCAAGATGAATGGA	GCAGCCTTGGTCTTAGACGCTACAA	For constructing RNAi-1 vector
SS1G_14097	RNAi1-14097 F/R	GGCTGATGGCTGCTTATCGTGCTGATAC	GTAGCAACTGGAAGGTGAACATTGGAGAAGATT	For constructing RNAi-1 vector
SS1G_02281	RNAi2-02281 F/R	CCATCGATGGATCCGCATCGGTCTATCAGCGGAGAAGGAG	CCCAAGCTTCTGCAGCCATCCAGTGTTCAACAACCAAGCATTAGC	For constructing RNAi-2 vector
SS1G_03827	RNAi2-03827 F/R	CCATCGATGGATCCAATGGAAGGAGATCAGCGAAGTTGTCAAGG	CCCAAGCTTCTGCAGGGCTAGTGATGTGAGACCAATCGTGCTT	For constructing RNAi-2 vector
SS1G_04568	RNAi2-04568 F/R	CCATCGATGGATCCGGATCAGAGATCAACATTACAACCGATGACAAGT	CCCAAGCTTCTGCAGAATCACCACGAGCAACCATGACACCAT	For constructing RNAi-2 vector
SS1G_06022	RNAi2-06022 F/R	CCATCGATGGATCCCGTCGTCGCCAACAAGAGTCCTGAAG	CCCAAGCTTCTGCAGATGGGTAGGAGTGGAAATTAAAGATGATGGGTTTG	For constructing RNAi-2 vector
SS1G_06400	RNAi2-06400 F/R	CCATCGATGGATCCAGCAGAAGACGGTGGTTATGGTGTTGT	CCCAAGCTTCTGCAGTCAGGAGTCGTGAGTAACCCTTCCAAATC	For constructing RNAi-2 vector
SS1G_06561	RNAi2-06561 F/R	CCATCGATGGATCCTGACAACAACTCCCTCTACACTCAACCA	CCCAAGCTTCTGCAGTTTCTCTCCCTCACGAACCCAGACAC	For constructing RNAi-2 vector
SS1G_10246	RNAi2-10246 F/R	CCATCGATGGATCCAAGATTCACGAGGCTATTCCAGAACTTGTTG	CCCAAGCTTCTGCAGGAAGGAGTGTGCTTGGAGGTCAATGC	For constructing RNAi-2 vector
SS1G_11433	RNAi2-11433 F/R	CCATCGATGGATCCTTCGTTGGAGGTAACTTCAAGATGAATGGA	CCCAAGCTTCTGCAGGCAGCCTTGGTCTTAGACGCTACAA	For constructing RNAi-2 vector
SS1G_14097	RNAi2-14097 F/R	CCATCGATGGATCCGGCTGATGGCTGCTTATCGTGCTGATAC	CCCAAGCTTCTGCAGGTAGCAACTGGAAGGTGAACATTGGAGAAGATT	For constructing RNAi-2 vector
SS1G_02281	qPCR-02281 F/R	GGTTCTGCCATCACATCTAC	CCATACTTCAGGAGTCATAGGT	For qRT-PCR
SS1G_03827	qPCR-03827 F/R	CCTCCAGTATGCCATCTTAG	CCTTAACGACCTTGTCTTCT	For qRT-PCR
SS1G_04568	qPCR-04568 F/R	CGTATCGCTGGTCTCAATGT	ACTTGTCATCGGTTGTAATGTTG	For qRT-PCR
SS1G_06022	qPCR-06022 F/R	GTCACAAGGAACTGGAATCT	GGACTACGAAGGACAATCAT	For qRT-PCR
SS1G_06400	qPCR-06400 F/R	GGCGGAGTGTGGAAGGATTA	GAATGTATTAGTGCCGTGGAGAAC	For qRT-PCR

Genes	Primer name	Forward primer (5’-3’)	Reverse primer (5’-3’)	Use
SS1G_06561	qPCR-06561 F/R	CATCCATCGCTGGTGGTATT	GCCGTGCTCCTTGAAGTATT	For qRT-PCR
SS1G_10246	qPCR-10246 F/R	CCTCGGTGATGGATGTCTGA	CGGCTTGGCACTTCTTGATAG	For qRT-PCR
SS1G_11433	qPCR-11433 F/R	TTCGTTGGAGGTAACTTCAAGATG	GCGTCCAGGTAATGTTGCTAT	For qRT-PCR
SS1G_14097	qPCR-14097 F/R	CTGTTCATCTCGGTGCTCTC	GCAAGTTCTTTCCATTGGTCTTT	For qRT-PCR
SS1G_01105	qPCR-01105 F/R	TGATTACATTACCGCAGACAACTT	CACCGCCACCGATAATAACAA	For qRT-PCR
SS1G_01844	qPCR-01844 F/R	ATGGAATGTTGGCAGGAATAGC	TTTCTAAGTTCAGCGACCTTTGG	For qRT-PCR
SS1G_02202	qPCR-02202 F/R	CATCACTACAACCACGAACCA	AACACATCTTCCTTCTCCAACAAT	For qRT-PCR
SS1G_06790	qPCR-06790 F/R	CATCAGTGCCGAAGTTCCA	GCCATCTCCTTATCATCCATACAA	For qRT-PCR
SS1G_07798	qPCR-07798 F/R	ATGTCTATGCGTGTTCCAACTG	GTCCTCGGTGTAAGCCAATATG	For qRT-PCR
SS1G_08408	qPCR-08408 F/R	AACTTCAACCTCGTCTCCTTAC	ACTCCGACTATTCTCTTCAATCTTC	For qRT-PCR
SS1G_08827	qPCR-08827 F/R	GTTACTGACGGTTCTCGCATT	GGAAGGATGAATACCAGCACAA	For qRT-PCR
SS1G_08975	qPCR-08975 F/R	TGTTGTTGCTGGTGCTTCTG	TCATCACGAGTCATTCCTGGTT	For qRT-PCR
SS1G_11369	qPCR-11369 F/R	TCAGAGGAAGAAGAAGAAGCCATT	AGATTGTGCCGACCGAGAC	For qRT-PCR
SS1G_12079	qPCR-12079 F/R	CGAAGACCACGAATACGAAGT	TCCTCAACCGAACATCTAATAACC	For qRT-PCR
SS1G_13825	qPCR-13825 F/R	CTTCAACACCAACGCATCAAT	TGACCACCAACAACAACAATG	For qRT-PCR
β-tubulin	Tub F/R	TTGGATTTGCTCCTTTGACCAG	AGCGGCCATCATGTTCTTAGG	For qRT-PCR
hph	Hyg F/R	GGATGCCTCCGCTCGAAGTA	TTGCAAGACCTGCCTGAAACCG	Transformants confirmation
